# Base-rate expectations modulate the causal illusion

**DOI:** 10.1371/journal.pone.0212615

**Published:** 2019-03-05

**Authors:** Fernando Blanco, Helena Matute

**Affiliations:** Departamento de Fundamentos y Métodos de la Psicología, Universidad de Deusto, Bilbao, Spain; New York Unviersity, UNITED STATES

## Abstract

Previous research revealed that people’s judgments of causality between a target cause and an outcome in null contingency settings can be biased by various factors, leading to causal illusions (i.e., incorrectly reporting a causal relationship where there is none). In two experiments, we examined whether this causal illusion is sensitive to prior expectations about base-rates. Thus, we pretrained participants to expect either a high outcome base-rate (Experiment 1) or a low outcome base-rate (Experiment 2). This pretraining was followed by a standard contingency task in which the target cause and the outcome were not contingent with each other (i.e., there was no causal relation between them). Subsequent causal judgments were affected by the pretraining: When the outcome base-rate was expected to be high, the causal illusion was reduced, and the opposite was observed when the outcome base-rate was expected to be low. The results are discussed in the light of several explanatory accounts (associative and computational). A rational account of contingency learning based on the evidential value of information can predict our findings.

## Introduction

Recent research has investigated the so-called "causal illusion" or "illusion of causality", a phenomenon that consists in believing that a causal relation exists between a potential cause, C, and an outcome, O, when they are causally unrelated but coincide frequently [1]. For instance, when a bogus medicine is used to treat a given symptom that disappears spontaneously very often, it is common to mistakenly believe that the remission of the symptom is caused by the medicine. Causal illusions have been typically identified in contingency learning experiments conducted in the laboratory, but they have been proposed to underlie many everyday superstitions and irrational beliefs [2–6], thus giving birth to a fruitful research field that taps into both theoretical and societal issues. Even in laboratory experiments, a relevant amount of evidence has been collected in computer tasks that used meaningful scenarios, such as the typical medicine-evaluation task, in which participants are asked to judge the effectiveness of a medicine in treating a fictitious disease. These experiments have revealed important information that can be used to alleviate the undesired effects of the causal illusion in real life situations, such as pseudomedicine usage or self-medication [7].

**Fig 1:**
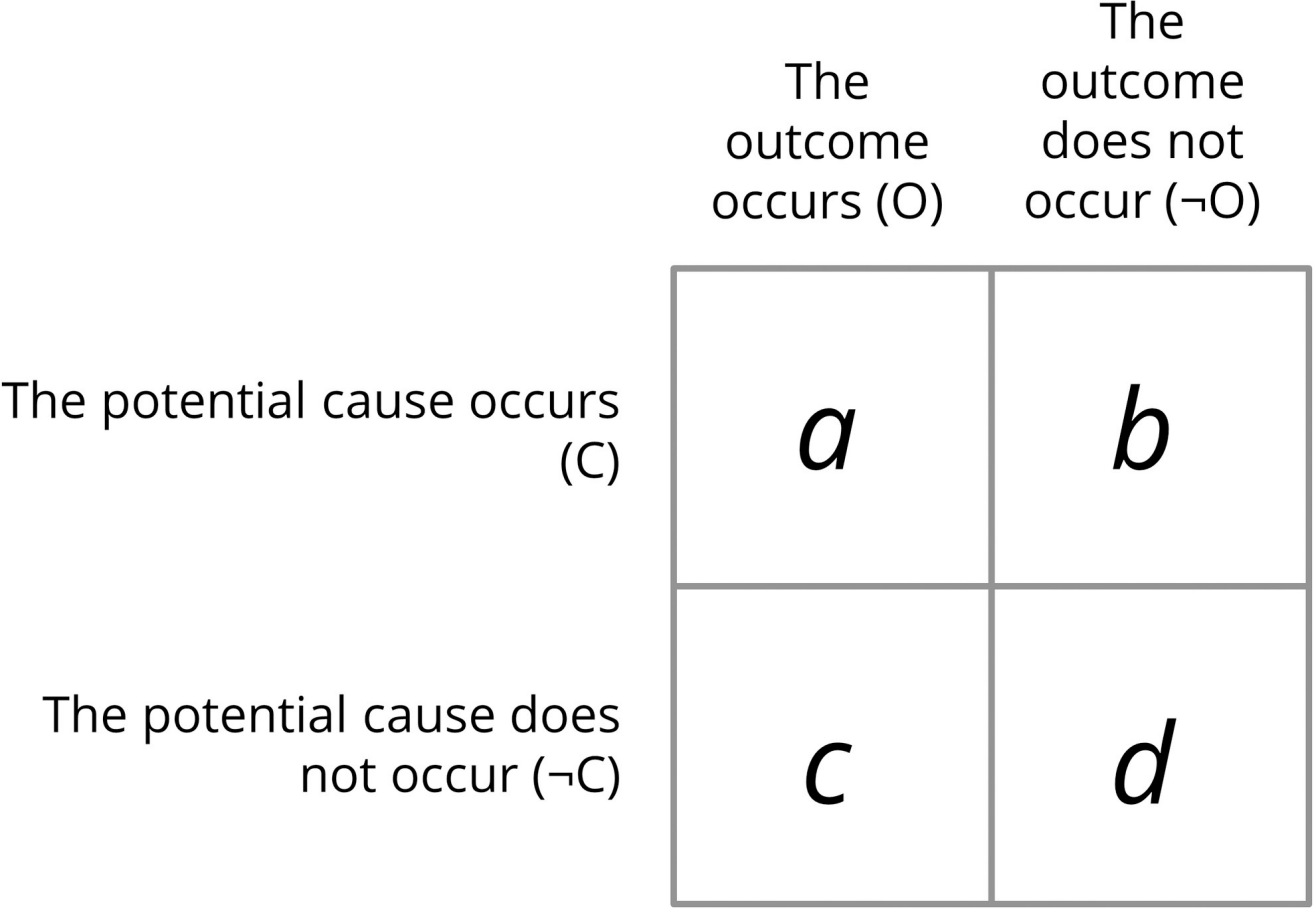
Contingency table for one potential cause and one outcome.

The experimental paradigm typically used to investigate causal illusions is the contingency learning task [8,9]. In this task, the participant is presented with a series of trials (e.g., medical records of fictitious patients) in which the cause C and the outcome O can either occur or not occur. Thus, there are four types of trials in the task, and they can be arranged in a contingency table like the one depicted in Fig 1: in type *a* trials, both the potential cause and the outcome occur; in type *b* trials, only the potential cause occurs; in type *c* trials, the outcome, but not the potential cause, occurs; finally, in type *d* trials, neither the potential cause nor the outcome occurs [10].

For a causal relation to exist (unless there is an uncontrolled external factor), the outcome must be contingent on the potential cause. Following our medical example, this means that, if the medicine is actually a cause of the remission of the disease, then the remission must be observed more frequently when the medicine is taken than when it is not taken. This reasoning is formalized in the widely used contingency index ΔP [8,10]:

$$\Delta P=P(O|C)-P(O|\neg C)=\frac{a}{a+b}-\frac{c}{c+d},$$ (1)

where P(O|C) and P(O|¬C) are the conditional probabilities of the outcome given the presence and the absence of the cause, respectively. The letters *a*, *b*, *c* and *d* are the frequencies of each of the four types of trials (see Fig 1) that can be used to compute the two mentioned conditional probabilities. The ΔP index takes on values between -1 and 1. The closer to zero this value is, the weaker the association between the potential cause and the outcome, and therefore the causal relation between them becomes implausible. When P(O|C) equals P(O|¬C), the contingency is null (i.e., ΔP = 0), and we conclude that the potential cause does not actually affect the probability of the outcome. Under some circumstances, however, people tend to believe that there is a causal relationship between the potential cause C and the outcome O, despite their contingency being null. That is, they develop a causal illusion [1].

One of the factors that are relevant for the formation of a causal illusion is the probability with which the outcome of interest occurs, P(O) (e.g., how often the symptoms of a disease remit). Even when there is no actual causal relation between a potential cause (e.g., a medicine) and an outcome (e.g., remission of the symptoms), high levels of P(O) lead to strong perceptions of causality, or causal illusions. This is sometimes called the outcome-density bias [11–13]. Thus, if a medicine is followed by the remission of the symptoms with high probability (e.g., seven in ten times), then it will seem to be an effective treatment, even if it is actually useless and the remissions occur just as frequently in its absence.

Additionally, some factors that are external to the experimental setting and precede the training session can also affect causal learning [14–16] and, therefore, may also modulate the causal illusion. Imagine the following situation, inspired by the usual medicine-evaluation task that is used in contingency learning experiments: You are a doctor who is testing a new medicine to treat common flu. You test the medicine on ten volunteers who are currently suffering from the flu. A few days after, three of them (i.e., 3 out of 10) feel better. Given this situation, would you be inclined to think that the new medicine is effective? We believe that most people would conclude that this medicine is not effective at all, because the rate of remissions when the medicine is used is rather low, that is, P(O|C) = .30 (three type *a* trials against seven type *b* trials).

Now consider a similar scenario, only changing the name of the disease: instead of treating common flu, now the fictitious doctor is treating cancer, and she gets the same results: three out of ten remissions when the medicine is used, that is, P(O|C) = .30. It is likely that, in this latter scenario, you would conclude that the medicine is highly effective. Why do we expect this difference between the two scenarios? After all, both decisions are based on the same observed information (i.e., success rate of 30%). Thus, the scenarios in this example differ only in the implicit assumptions that people make. When we approach the common flu scenario, our previous experience with the world dictates that we should expect a high rate of spontaneous remissions (much higher than 30%): almost everybody who catches a flu ends up feeling better after a few days. Therefore, the actual observed rate of remissions when using the medicine is actually lower than the expected base-rate, suggesting that the medicine is not working well. On the other hand, when we are presented with the cancer scenario, our assumptions are different: we know that it is highly unlikely that cancer remits spontaneously. That is, in the cancer scenario, we assume that the spontaneous remission base-rate is very low, much lower than 30%. Hence, the observed remission rate when using the treatment is much higher in comparison, suggesting that the medicine works. In sum, what makes these two scenarios so strikingly different is the previous knowledge (and expectations) about their respective outcome base-rates, or P(O|¬C). A piece of information that was deliberately absent from the description of the problem and, thus, is assumed spontaneously by the reader.

In these two examples, previous knowledge about outcome base-rate affects the way people interpret the available information. Current theories about contingency learning that are used to model causal illusions provide mechanisms that could accommodate the effect of the base-rate expectations that we have just illustrated. One of the ways in which this can be achieved is by means of cell-weighting mechanisms. That is, by giving more importance to some pieces of information (i.e., each of the cells in Fig 1) than to others. For example, in the case of the medicine for the flu (first scenario), because we know that the outcome base-rate, P(O|¬C), is high, we would expect being exposed to a lot of fortuitous outcome occurrences, and consequently grant these cells (*a* and *c*) less importance. This means that we would not be impressed enough by the rather small number of type *a* trials (3 out of 10) because they could be attributed to chance. In the second scenario, by contrast, the expectation of a low P(O|¬C) (i.e., patients suffering from cancer rarely recover from the disease without a treatment) would make outcome occurrences (cells *a* and *c*) very salient, because we expect that they would appear with extremely low probability. Therefore, the scarce type *a* trials cannot be interpreted as mere coincidences that result from chance. Rather, they would be important enough to judge the medicine as effective. In fact, a rational, Bayesian analysis of causal induction concludes that, whenever a learner knows or expects that the cause and the outcome occur with low probability (as in the cancer scenario), then he or she should weight type *a* trials more heavily than the rest of them when making a causal judgment [17].

In this article, we report two experiments that aimed to reveal how the expectations about the outcome base-rate, P(O|¬C), affect the subsequent contingency learning phase, modulating the causal illusion. The predictions follow the same rationale as the example provided above (common flu vs. cancer) and align with the optimal Bayesian reasoning account by McKenzie & Mikkelsen [17]. In addition to the usual judgment of causality that is used to assess the causal illusion, we included a novel type of question (evidential value question) to investigate the extent to which each type of trial (*a*, *b*, *c* and *d*) was given importance to judge the causal relation. The experiments reported here resemble others in the literature [18], although our aims are different: Here we focus on the causal illusion, and consequently we deal with null contingencies only, and compare between different assumed base-rates (a test that was absent in the cited article).

## Ethics statement

The procedure used in these two experiments was examined and approved by the Ethical Review Board of the University of Deusto. The participants were informed before the experiment that they could quit the study at any moment by closing the browser window. The data collected during the experiment were sent anonymously to the experimenter only upon explicit permission by the participant, indicated by clicking on a "Submit" button. If the participant clicked on the "Cancel" button, the information was erased. No personal information (i.e., name, IP address, e-mail) was collected. We did not use cookies or other software to covertly obtain information from the participants.

## Experiment 1

In this experiment, we used the standard contingency learning task with a completely new cover story in which participants had no previous experience and, thus, no clear expectations. Half of our participants were exposed to a pretraining phase in which they learned that the outcome base-rate was high (High base-rate group). The other half of participants was not pretrained (Control group). Then, after the usual training phase showing a null contingency between the potential cause and the outcome that was identical for all participants, we assessed the causal illusion in both groups. We expected the High base-rate group to show little or no causal illusion compared to the Control group. In sum, this experiment represents the "flu scenario" described in the example above, in which the outcome base-rate is assumed to be high.

### Method

#### Participants and apparatus

Sixty-five anonymous Internet users took part in the study through our virtual laboratory website [http://www.labpsico.deusto.es/]. The computer program randomly assigned each participant to one of two groups, a High base-rate group (*n* = 36) and a Control group (*n* = 29). The sample size was decided after running an unpublished pilot study with similar design (but a different cover story), on a smaller sample (*N* = 26), which produced nonsignificant results in the expected direction. To achieve enough power, we decided to at least double the number of participants used in the pilot study. The experiment was programmed in *JavaScript*, a web-based language that is interpretable by most browsers.

#### Procedure

The experiment was a modified version of the standard contingency learning task that we have used previously to study causal illusions [1]. The procedure, materials and data are publicly available at the Open Science Framework [19]. Participants were instructed to imagine that they were scientists working on a distant-future space colony, under the threat of a race of evil aliens. According to the instructions (available online [19]), some of the aliens had an armored skin resistant to heavy laser weapons, and thus were extremely dangerous. However, other aliens had a mutation (called "XG Vulnerability") that impaired the development of their armor. Therefore, the mutation made these aliens vulnerable to humans' weapons. Participants were told that the mutation appeared naturally in the alien population. To further study the aliens' development and physiology, a number of alien eggs were captured. This sci-fi setup was aimed at suggesting to the participants that the outcome of interest (i.e., the mutation) occurred spontaneously with a given, unspecified, base-rate.

The crucial difference between the High base-rate group and the Control group was the presence of a pretraining phase in the former to induce the base-rate expectations. After the instructions were given, participants in the High base-rate group were presented with a sample of different alien eggs. On each trial, the picture of an alien egg and its alphanumeric label (a randomly generated string, e.g., "*Egg AV35*") were displayed on the top panel of the screen. The goal of the participant was to predict whether or not the current egg would present the mutation "XG Vulnerability" by clicking on one of the two available buttons (labeled "It will develop the XG Vulnerability" and "It will NOT develop the XG Vulnerability"). Immediately after making this prediction, the outcome information was displayed on the bottom panel of the screen. If the current alien egg had the mutation, a picture of an alien and a shield crossed out in red was displayed, together with the sentence "The alien has the XG Vulnerability". In the opposite case, a picture of the alien and the sentence "The alien does NOT have the XG Vulnerability" were presented. After a delay of one second, a button labeled "Next egg" was available to proceed to the next trial.

A series of 20 pretraining trials were delivered in the High base-rate group. Fourteen eggs gave birth to mutants showing the vulnerability, that is, the base-rate of the outcome was high (.70). At the end of the sequence of 20 trials presented in random order, the screen showed a sentence highlighting the high base-rate: "As you have seen, the number of eggs resulting in mutant aliens with the XG Vulnerability is very high". This was a means to ensure that the base-rate expectation manipulation worked.

Immediately after learning this information, the next screen asked about the base-rate of the mutations by means of a base-rate judgment: "Imagine you see 100 alien eggs. Of these 100, how many of them would give birth to aliens with the XG Vulnerability mutation?". The question was answered by clicking on a numerical scale from 0 to 100. In the Control group, this judgment was collected without any pretraining (immediately following the instructions), which means that they had no informed reasons to prefer one value over others. Thus, the base-rate judgment served as a means to check whether or not the pretraining phase worked as intended to install high base-rate expectations in the high-base-rate group.

After collecting the base-rate judgment, all participants were exposed to the contingency training phase, which was identical in both groups, and similar to the "active" version of the contingency learning task we used in previous experiments [20]. The instructions to this training phase made clear to the participants that their goal was to test the effectiveness of a newly-developed mutagenic agent that, when sprayed on the unhatched eggs, might increase the chances to give birth to a mutant alien with the XG Vulnerability. To this end, the participants saw a series of different eggs (each one labeled with a unique alphanumeric code). This information was presented on the top panel of the screen. Then, the participants had the opportunity to decide whether or not to apply the agent to the egg for testing purposes. This was indicated in the middle panel with the sentence "You can use the mutagenic agent now (by pressing the spacebar)" and a picture of a test tube filled with a green liquid. This information stayed on the screen for three seconds during which the participants had to make their decision. After the three seconds passed, the panel showed the corresponding feedback: if the participant pressed the spacebar, then the sentence "You have used the mutagenic agent" and a picture of the test tube pouring the liquid appeared; if the participants did not press the spacebar, then the sentence "You have not used the mutagenic agent" and the same picture crossed out in red were presented. After one second, the information about the outcome occurrence was displayed on the bottom panel: if the current egg gave birth to a mutant alien (outcome-present trial), then the picture of an alien with a shield crossed out in red appeared accompanied by the sentence "The alien has the XG Vulnerability"; otherwise (outcome-absent trial), the picture of the alien appeared together with the sentence "The alien does NOT have the XG Vulnerability". Below this information, a button labeled "Continue" was available to proceed to the next trial. In the contingency training phase, the sequence contained 50 trials, out of which 35 showed an alien with the XG Vulnerability mutation, that is, P(O) was .70. During the training phase, the eggs produced mutants with the same probability (i.e., .70) regardless of the participants' decisions, which means that the mutagenic agent was completely ineffective. Importantly, this is the same probability of the outcome that was used in the pretraining phase of the High base-rate group. The order in which mutant and non-mutant aliens appeared during the session was randomized for each participant.

After the 50 eggs were presented, the participants were asked to answer several questions. The first one was a causal judgment, with a wording similar to the one we used in previous studies [2,3]: "To what extent do you think that the mutagenic agent was effective to produce aliens with the XG Vulnerability?". This was answered on a scale from 0 (labeled "Not effective at all") to 50 ("Moderately effective") to 100 ("Perfectly effective"). Following the causal judgment, we asked a confidence judgment: "To what extent are you sure about your previous answer?", on a scale from 0 ("Not sure at all") to 100 ("Completely sure"). This was aimed at capturing the uncertainty in the causal estimation.

The next two questions concerned the perceived probabilities of the outcome, conditional on the presence/absence of the potential cause, i.e., P(O|C) and P(O|¬C), and were presented in random order for each participant. They were worded in terms of frequencies rather than of probabilities, to make them easier to understand by participants [21]. That is, for the P(O|C) question, "Imagine you are presented with 100 more eggs, and that the mutagenic agent is used on all of them (i.e., 100). Out of these 100 eggs, how many of them do you think would give birth to aliens with the XG Vulnerability?"; and for the P(O|¬C) question, "Imagine you are presented with 100 more eggs, and that the mutagenic agent is used on none of them (i.e., 0). Out of these 100 eggs, how many of them do you think would give birth to aliens with the XG Vulnerability?". Both questions were answered on a numeric scale from 0 to 100.

Finally, we collected a new measure called “evidential value questions”. Four screens were presented in random order for each participant, preceded with a screen with the sentence "Now observe the following records from the study". Each record contained the information of one type of trial (i.e., *a*, *b*, *c*, and *d*). That is, in the screen corresponding to a type *a* trial, the mutagenic agent was used and the alien developed the mutation; in the screen corresponding to a type *b* trial, the mutagenic agent was used but the alien showed no mutation, and so on. Below this information, a question was displayed. For type *a* and type *d* trials, the question read: "Please, indicate whether you think that the result of this record is due to MERE CHANCE or is COMPELLING EVIDENCE in favor that the mutagenic agent helps to produce aliens with the XG Vulnerability". For type *b* and type *c* trials, the question read: "Please, indicate whether you think that the result of this record is due to MERE CHANCE or is COMPELLING EVIDENCE against that the mutagenic agent helps to produce aliens with the XG Vulnerability". The question was answered by clicking on one of the available buttons, labeled "coincidence" or "evidence". The questions corresponding to the four types of trials (*a*, *b*, *c*, and *d*) were presented in these questions in random order. The aim of these questions was to explore potential differences between the groups in the informational value attributed to each type of trial.

### Results and discussion

#### Judgments

All the data collected in Experiment 1 and Experiment 2 are available at the Open Science Framework [19]. Table 1 contains the descriptive statistics for the five types of judgment collected (Base-rate, Causal, Confidence, and the two conditional probabilities). No heteroscedasticity problems were detected (all Levene’s tests *p* > 0.55). However, since the data of some of the judgments are not normally distributed (e.g., base-rate judgments in the pretrained groups in both experiments), we used robust estimation by adopting the Yuen’s test [22,23], based on trimmed means (trim proportion was set to default, 0.20), which compensates for this problem at the cost of statistical power. Both standard (non-corrected) and robust analyses are reported for completeness, and they produce similar results.

**Table 1:** Descriptive statistics of the five judgments collected in Experiment 1.

	Control group			High Base-rate group		
Judgment	*M*	*SD*	95% CI	*M*	*SD*	95% CI
Base-rate	39.55	15.64	[33.86, 45.24]	64.33	15.89	[59.14, 69.53]
Causal	50.62	23.68	[42.00 59.24]	33.06	28.76	[23.66, 42.45]
Confidence	70.59	25.98	[61.13, 80.04]	62.83	28.84	[53.41, 72.26]
P(O|C)	52.41	23.47	[43.87, 60.95]	61.89	21.58	[54.84, 68.94]
P(O|¬C)	60.00	19.64	[52.85, 67.15]	61.00	20.49	[54.31, 67.69]

First, we ensured that the pretraining phase led participants to develop high expectations about the outcome base-rate (i.e., we conducted a manipulation-check). Base-rate judgments were significantly higher in the High base-rate group than in the Control group, *t*(63) = 6.293, *p* < 0.001, *d* = 1.57 [Yuen’s test: *t*(33.0) = 4.96, *p* < 0.001, ξ = 0.74]. In other words, the manipulation worked as intended to encourage higher expectations about the outcome base-rate. In fact, the mean base-rate judgment in the pretrained group was very close to the normatively expected value assuming that participants start from a uniform prior: that is, before pretraining, the prior on the base-rate could be uniform, Beta (1, 1). Updating this prior with the observed data (14 mutants, 6 non-mutants) yields a posterior with Beta (1+14, 1+6), whose mean is 0.68, close to the mean value (one-sample t-test, *p* = 0.90). In the Control group, however, the judgments were significantly lower than 50 (*p* = 0.001), which suggests that lay people with have no additional information intuitively assign a relatively low base-rate to the occurrence of mutations [24]. As long as there is significant difference between the groups in the expected direction, the manipulation can be considered successful.

**Fig 2:**
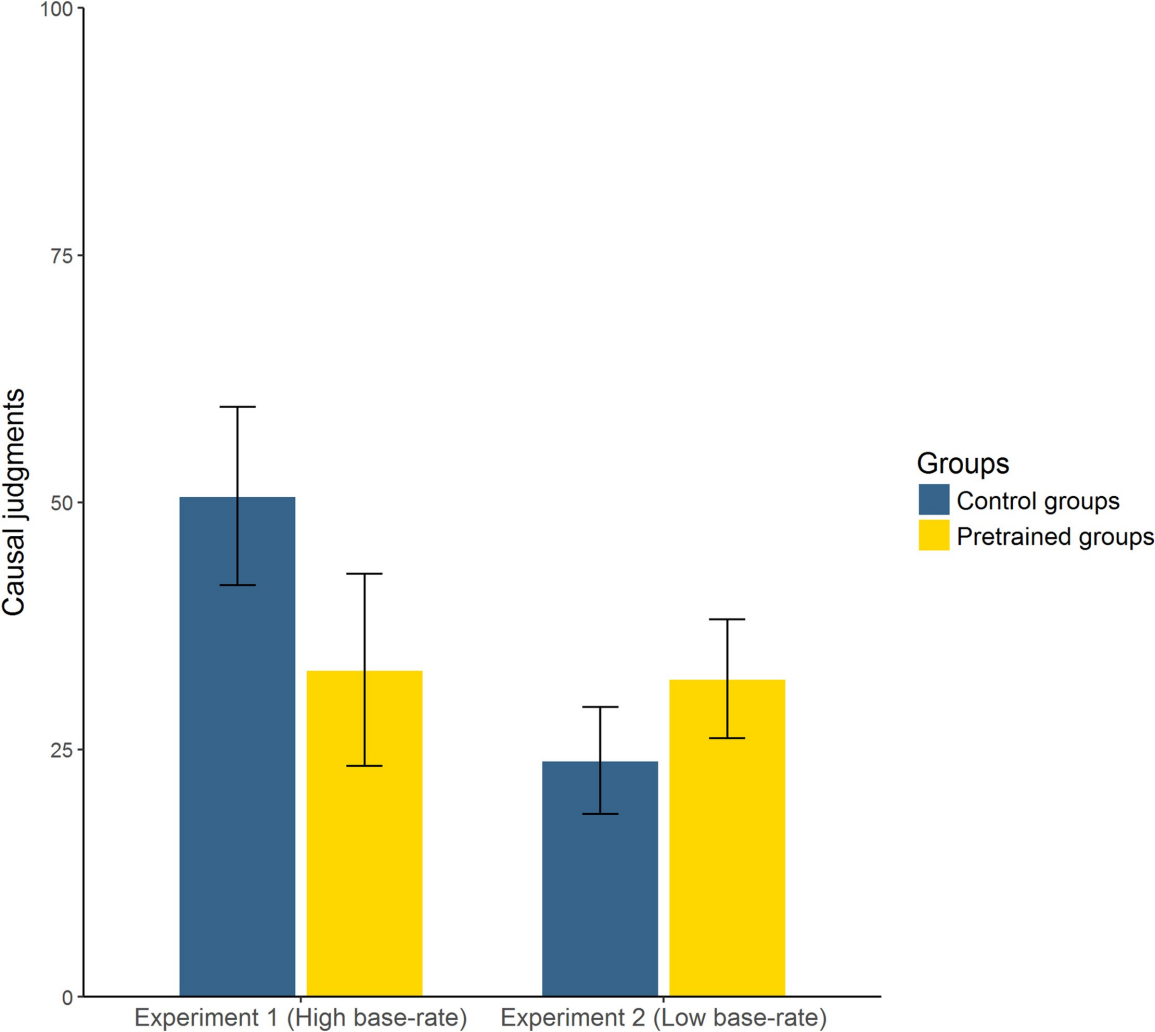
Mean causal judgments in each group of Experiment 1 (high base-rate of the outcome) and Experiment 2 (low base-rate of the outcome). Error bars represent 95% CIs for the mean.

Causal judgments were our main dependent variable. They are depicted in Fig 2. As we expected, participants in the High base-rate group gave lower causal judgments than did participants in the Control group, *t*(63) = 2.644, *p* = 0.01, *d* = 0.66 [Yuen’s test: *t*(38.1) = 3.10, *p* = 0.004, ξ = 0.48]. This difference cannot be attributed to differences in their confidence, because the two groups did not differ significantly in their confidence judgments (see Table 1), *t*(63) = 1.125, *p* = 0.265, *d* = 0.28 [Yuen’s test: *t*(36.4) = 0.755, *p* = 0.455, ξ = 0.14]. In sum, these were the main results we predicted.

Next, we examine the answers given to the two conditional probability questions (Table 1), which were collected with exploratory aims. The analyses revealed no significant between-group differences for the P(O|C) question, *t*(63) = 1.692, *p* = 0.096, *d* = 0.42 [Yuen’s test: *t*(30.6) = 1.94, *p* = 0.062, ξ = 0.32], and for the P(O|¬C) question, *t*(63) = 0.199, *p* = 0.843, *d* = 0.05 [Yuen’s test: *t*(37.9) = 0.61, *p* = 0.546, ξ = 0.10].

In addition, we used the conditional probability questions to compute a perceived ΔP index (as Eq 1 implies, it is the subtraction of the two conditional probability questions) that could indirectly inform us about the perceived causality. This variable did not differ between groups, *t*(63) = 1.197, *p* = 0.236 [Yuen’s test: *t*(24.4) = 1.55, *p* = 0.133, ξ = 0.265], showing values close to zero (in the Control group, *M* = -0.0759, *SD* = 0.327; and in the High Base-rate group, *M* = 0.009, *SD* = 0.244). Furthermore, the ΔP index computed from the conditional probability questions did not correlate with causal judgments in either group (*r* = -0.017, *p* = 0.932, and *r* = -0.29, *p* = 0.086, respectively). Taken together, these results could suggest that people do not combine the two pieces of the conditional probability information to obtain the causal judgment, although we must remain cautious as the experiment was not designed for this purpose. The same analyses were conducted on the predictions by the Power PC model for generative relationships [25], with identical (non-significant) results.

Finally, we have conducted simulations to study the process of Bayesian update of the base-rate knowledge from the prior distribution (before any information is given) to the posterior distribution, in light of the data provided during the pretraining and training phases. We used beta distributions to model the belief update process, by means of the MASS package [26,27] for R. These analyses are described in detail in the S1 Appendix file.

#### Probability of the cause

Because we know from previous studies that the probability of the cause, P(C), biases causal and contingency estimations [28–31], and that previous knowledge and expectations can indeed affect the contingency judgment by actively biasing behavior [32], we examined the possibility that the two groups differed in this variable. The P(C) was computed as the proportion of trials in which the participant decided to use the mutagenic agent during the training phase: in the High base-rate group, *M* = .60, *SD* = .26, 95% CI [.51, .68]; and in the control group, *M* = .63, *SD* = .23, 95% CI [.55, .72]. There were no significant differences in P(C) between the groups, *t*(63) = 0.563, *p* = 0.575, *d* = 0.14 [Yuen’s test: *t*(31.80) = 0.482, *p* = 0.633, ξ = 0.08]. Therefore, this potential confounding variable was controlled for. That is, the significant between-group differences in the causal judgments that we reported above cannot be attributed to between-group differences in the overall level of P(C).

**Fig 3:**
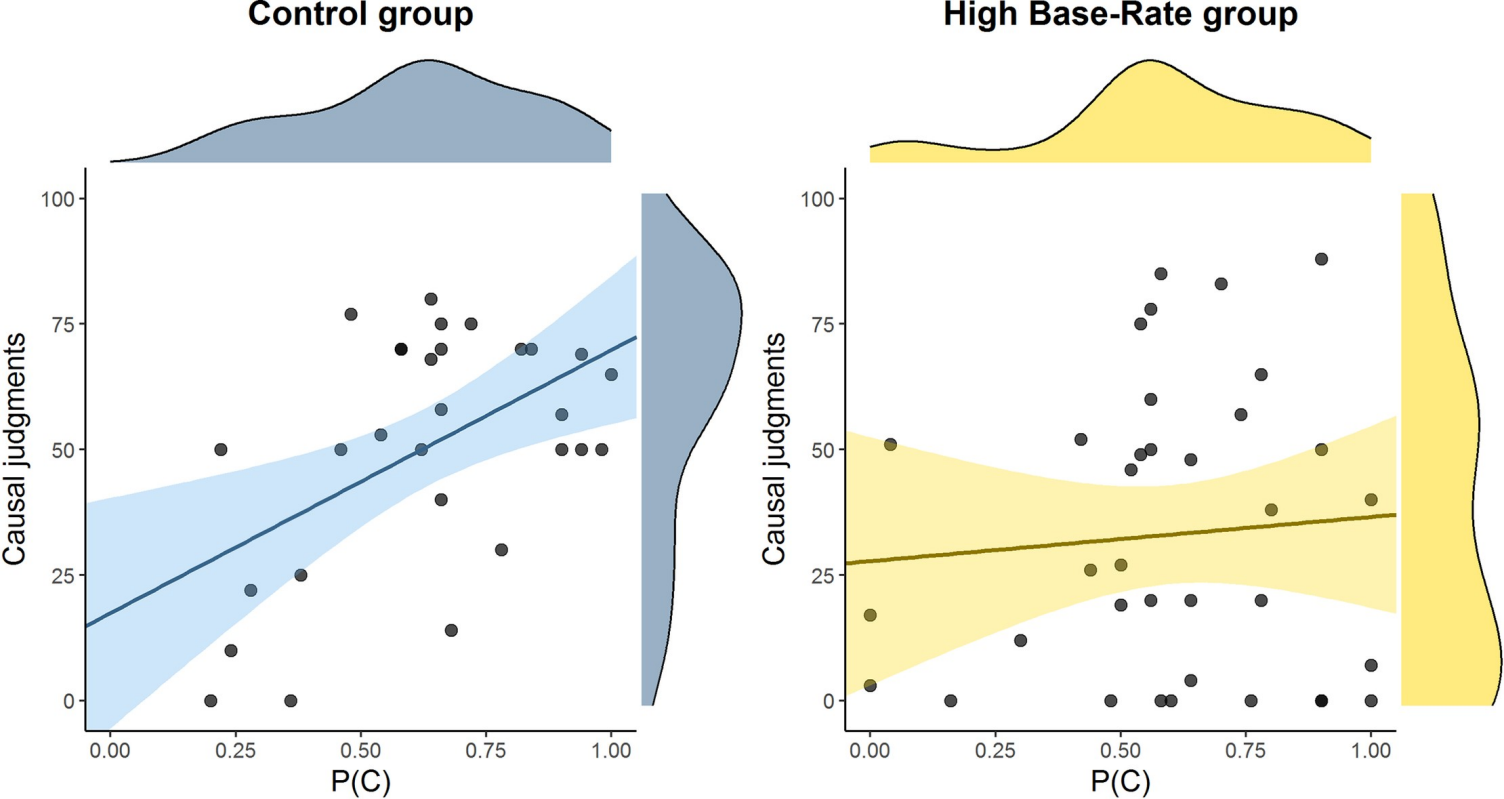
Scatter plot depicting the relation between P(C) and causal judgments in the two groups of Experiment 1, including marginal densities. Shaded areas represent 95% CIs for the regression slopes.

Additionally, the scatter plot in Fig 3 indicates that P(C) did increase the causal judgments in the Control group, β = .52, *t*(27) = 3.16, *p* = .004. This result, sometimes known as the “cause-density bias”, has been found recurrently in the literature [30,33]. On the other hand, the slope was not significantly different from zero in the High base-rate group, β = .08, *t*(34) = 0.47, *p* = .64. However, these conclusions must be taken with caution, as the slopes in the two groups did not differ significantly from each other, according to the Fisher’s transformation test (they were only marginally significant): *Z* = 1.88, *p* = 0.060.

Since participants were allowed to respond freely during the training phase, there could be individual differences in the overall contingency experienced by each participant [28,31] that might explain the pattern found in the judgments. Thus, we used the trial-by-trial data to compute the contingency to which each participant was actually exposed, according to two popular models, ΔP and Power PC (generative version), as seen in Table 2.

**Table 2:** Indexes obtained for the two models, ΔP and Power PC, computed from the actual training data in Experiment 1. The models cannot be computed for some participants (due to division by zero errors), so these cases are removed.

		Control group				High Base-rate group		
Model	*n*	*M*	*SD*	95% CI	*n*	*M*	*SD*	95% CI
ΔP	28	0.06	0.19	[-0.02, 0.13]	31	-0.03	0.15	[-0.09, 0.02]
Power PC	27	-0.05	0.48	[-0.14, 0.24]	31	-0.37	0.93	[-0.71, -0.03]

The actual ΔP and Power PC values experienced by participants did not correlate with the causal judgments in either group (all *p*s > 0.086), which is further evidence that participants are not following the normative rules in this null contingency setting.

#### Evidential value questions

**Fig 4:**
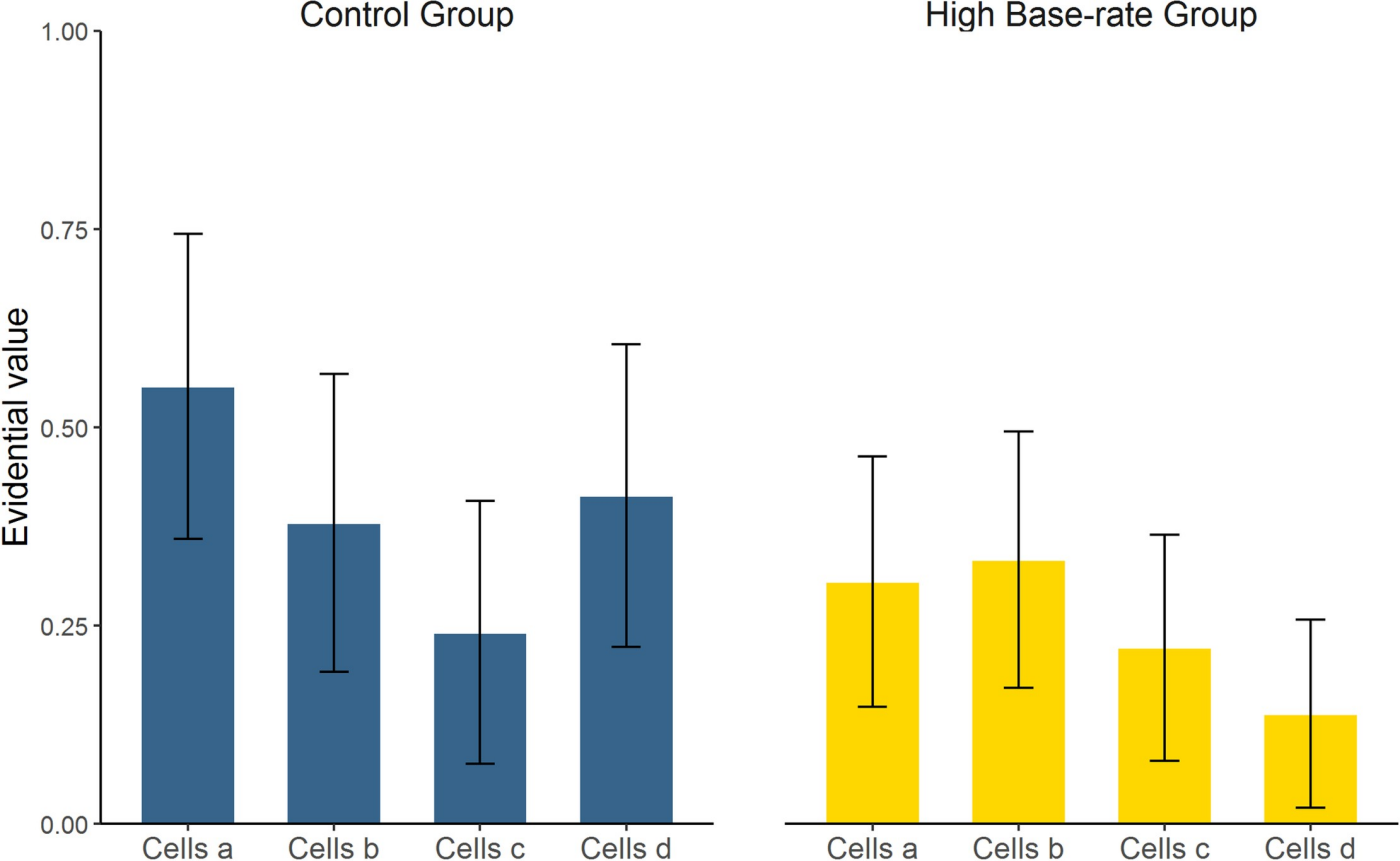
Mean proportion of “evidence” answers to the evidential value questions in Experiment 1, by type of trial: cells *a*, *b*, *c*, and *d*. Error bars depict 95% CIs for the mean.

The last variable in being examined was the answer given to the evidential value questions, summarized in Fig 4. This was an exploratory measure to collect information about intuitive cell-weighting mechanisms in our experiment. Participants were asked to categorize each trial type (*a*, *b*, *c*, and *d*) as either “coincidence” or “evidence”.

These data were analyzed by means of a GLM with logit link function for Binomial distributions (as the responses were either 0, “coincidence”, or 1, “evidence”). Group, Type of trial and their interaction were introduced as fixed factors, with random intercept at the subject level. The model converged at AIC = 302.6, with no significant effects (all *p*s > 0.063). Table 3 contains the marginal estimated means for each variable (converted to logit scale, where positive numbers indicate tendency to answer “evidence”). In the Control group, cells *c* significantly tended to be treated as a “coincidence”. In the High base-rate group, this happened to all trial types except *b*. Post-hoc contrasts revealed that no trial type differed between the two groups (all *p*s > 0.093). In sum, the results from this novel dependent variable were inconclusive.

**Table 3:** Evidential value questions in Experiment 1: Marginal estimated means (logit scale).

Group	Trial Type	Marginal estimated mean	95% CI
Control	a	0.307	[-0.704, 1.318]
	b	-0.608	[-1.645, 0.428]
	c	-1.454	[-2.583, -0.325]
	d	-0.419	[-1.444, 0.606]
High base-rate	a	-0.986	[-1.932, -0.041]
	b	1.380	[-1.759, 0.102]
	c	1.544	[-2.524, -0.496]
	d	-0.161	[-3.336, -1.026]

## Experiment 2

Experiment 1 examined the effect of high base-rate expectations on the subsequent development of a causal illusion. As we planned, expecting that the outcome would occur with high probability prevented (or reduced) the illusion, compared to a group in which this expectation was not pretrained. This was similar to the first situation presented as an example in the Introduction, in which observing a high rate of remissions when using a medicine for common flu did not lead to the conclusion that the medicine worked.

In Experiment 2, we used exactly the same procedure, but with a low outcome base-rate. That is, the situation in Experiment 2 was similar to the second scenario presented in the Introduction, in which a doctor is likely to believe that a treatment for cancer is working in spite of a relatively low remission rate when the medicine is used. Accordingly, we expected that, in Experiment 2, our participants would increase their causal illusion after a low base-rate pretraining. The Control group (without pretraining), in line with previous reports in which low outcome-density conditions were used, should show little or no overestimation of causality [13,34]. Thus, Experiment 2 complements Experiment 1 because it extends the predictions to a low, rather than high, outcome base-rate.

Additionally, Experiment 2 allows us to examine an alternative explanation for the results in Experiment 1. Since the pretraining phase is a series of cause-absent trials, participants in the pretrained (high base-rate) group would be overall exposed to a lower proportion of cause-present trials, or P(C), if we take into account the whole experimental session. A growing literature indicates that high P(C) is one of the factors that promote the illusion (i.e., the so-called “cause-density bias”) [30,31,35]. Thus, the lower judgments found in the High base-rate group in Experiment 1 could be due to participants experiencing an overall lower exposure to cue-present trials, if we assume that participants integrated both pretraining and training phases into one experience. Note that in Experiment 2, however, this potential explanation would lead to *the opposite prediction* to that we expressed before for this experiment. That is, in Experiment 2, the Control (i.e., not pretrained) group would still be exposed to proportionally more cause-present trials compared to the pretrained Low base-rate group, which would lead to higher judgments according to the prediction of the cause-density effect, but our prediction, based on low expected base-rates, is that the pretrained group should exhibit stronger illusions than the control group, due to the higher weight given to outcome-present trials, as they are assumed to be rare. Therefore, we could use Experiment 2 to discard an explanation for the results based on the cause-density bias.

### Method

#### Participants and apparatus

Since, based on previous literature, we expected that observing the causal illusion in a low outcome-density setting would be more difficult than it was in high outcome-density settings, we decided to at least double the number of participants used in Experiment 1. We recruited 132 participants through our website. Two of them were excluded from the analysis because of a data collection error. Thus, the final sample consisted of *N* = 130 participants: 65 in the Control group and 65 in the Low base-rate group.

#### Procedure

The procedure was identical to that used in Experiment 1, with the following two differences. First, the Low base-rate group was pretrained to expect the outcome with a low base-rate. That is, during the pretraining phase in this group, 6 out of 20 eggs gave birth to mutant aliens with the XG vulnerability, hence P(O) = .30. Second, during the training phase, all participants were exposed to the same low P(O): only 15 out of 50 eggs gave birth to mutant aliens (i.e., 30%).

### Results and discussion

#### Judgments

All the data collected in Experiment 1 and Experiment 2 are available at the Open Science Framework, [19]. Table 4 shows the descriptive statistics for the judgments in Experiment 2. Base-rate judgments were significantly lower in the Low base-rate group than in the Control group, *t*(128) = 6.00, *p* < 0.001, *d* = 1.05 [Yuen’s test: *t*(58.7) = 9.04, *p* < 0.001, ξ = 0.73]. This indicates that the pretraining phase successfully lowered the outcome base-rate expectations. As in Experiment 1, in fact the base-rate judgments in the pretrained group were very close to the normatively expected value if one assumes a uniform prior, that is, a posterior Beta (1+6, 1+14) yields a mean of 0.32. Still, base-rates seemed slightly underestimated, because in the Control group the judgments were lower than 50, *p* = 0.001, and in the Low base-rate group they were lower than 32. This aligns with Experiment 1 in suggesting that people systematically assume a low prior on the base-rate.

**Table 4:** Descriptive statistics of the five judgments collected in Experiment 2.

	Control group			Low Base-rate group		
Judgment	*M*	*SD*	95% CI	*M*	*SD*	95% CI
Base-rate	40.05	15.34	[36.32, 43.77]	26.20	10.53	[23.64, 28.76]
Causal	23.91	21.82	[18.60, 29.21]	32.15	24.34	[26.24, 38.07]
Confidence	57.29	29.20	[50.19, 64.39]	52.20	30.41	[44.81, 59.59]
P(O|C)	39.25	20.01	[34.38, 44.11]	37.58	20.28	[32.66, 42.51]
P(O|¬C)	38.80	20.79	[33.75, 43.85]	40.05	24.19	[34.17, 45.93]

Causal judgments of the two experiments are shown in Fig 2. Although the mutagenic agent was completely useless in both groups, it was perceived as more effective in the Low base-rate group, *t*(128) = 2.03, *p* = 0.044, *d* = 0.36 [Yuen’s test: *t*(75.3) = 1.92, *p* = 0.045, ξ = 0.242], in line with our prediction. That is, our manipulation increased the causal illusion in a condition, low P(O), in which it is typically weak.

No differences were found in the confidence judgments that were requested immediately afterwards, *t*(128) = 0.97, *p* = 0.332, *d* = 0.17 [Yuen’s test: *t*(74.0) = 1.03, *p* = 0.305, ξ = 0.132, which suggests that participants were similarly confident in their causal ratings in the two groups (see Table 4). This was also found in Experiment 1.

The participants' estimations of the conditional probabilities were fairly similar for both questions in the two groups, a result that also aligns with the previous experiment. Thus, we found no between-group differences for P(O|C) question, *t*(128) = 0.470, *p* = 0.639, *d* = 0.08 [Yuen’s test: *t*(75.3) = 0.839, *p* = 0.404, ξ = 0.110], and for the P(O|¬C) question, *t*(128) = 0.315, *p* = 0.753, *d* = 0.05 [Yuen’s test: *t*(72.3) = 0.833, *p* = 0.408, ξ = 0.125].

As in Experiment 1, we computed the perceived ΔP index as the difference between the two conditional probability questions. Again, this variable did not differ between groups, *t*(128) = 0.710, *p* = 0.479 [Yuen’s test: *t*(58.5) = 0.462, *p* = 0.646, ξ = 0.07], showing values close to zero (in the Control group, *M* = 0.004, *SD* = 0.217; and in the Low base-rate group, *M* = -0.024, *SD* = 0.249). The perceived ΔP index did not correlate with causal judgments: *r* = -0.132, *p* = 0.296, and *r* = -0.052, *p* = 0.679, in the Control and Low base-rate groups, respectively. The same analyses were also conducted on the predictions by Power PC model, with identical results, and consistent with those from Experiment 1. In sum, it seems that participants do not use either of these rules to produce their causal judgments.

Finally, simulations of the Bayesian belief update process are described in the S1 Appendix file. By modeling the process with beta distributions, we show how the knowledge of the base-rate is updated in light of the information given in the pretraining and training phases.

#### Probability of the cause

As in Experiment 1, we computed the proportion of trials in which the participants decided to use the mutagenic agent, that is, the probability of the cause, or P(C). This probability was significantly higher in the Low base-rate group (*M* = .70, *SD* = .184, 95% CI [.66, .74]) than in the Control group (*M* = .62, *SD* = .216, 95% CI [.57, .67]), *t*(128) = 2.425, *p* = .017, *d* = .04 [Yuen’s test: *t*(76) = 3.19, *p* = 0.002, ξ = 0.38].

**Fig 5:**
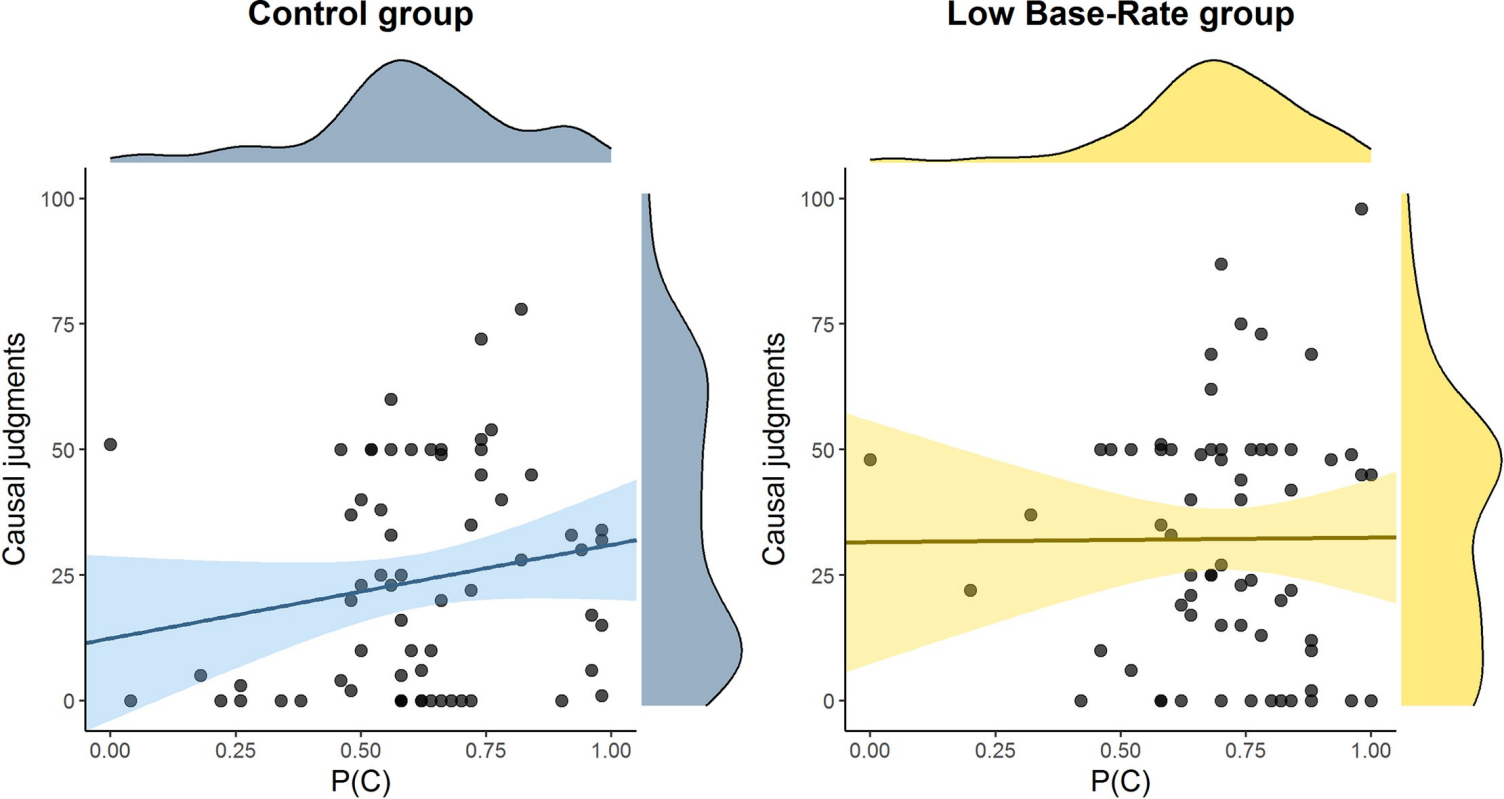
Scatter plot depicting the relation between P(C) and causal judgments in the two groups of Experiment 2, including marginal densities. Note that the fitted regression lines are relatively flat in both groups, suggesting that the two variables are not linearly related (see main text). Shaded areas represent 95% CIs for the regression slopes.

Fig 5 suggests that this difference does not challenge the conclusions derived from our main dependent variable, the causal judgments. As a simple regression analysis shows, P(C) was not a significant predictor of the causal judgments in either group, which means that this variable did not play a role in explaining the differences in the judgments: β = .18, *t*(63) = 1.49, *p* = .14 (Control group); β = .01, *t*(63) = 0.05, *p* = .96 (Low base-rate group). This contrasts with Experiment 1, in which we found (at least in the Control group) a significant effect of P(C), or cause-density bias, indicating that those participants with higher rates of responding gave higher judgments. That is, in a high P(O) setting (Experiment 1), we found a cause-density bias, but it was absent in the low P(O) setting (Experiment 2). The finding that P(C) effects do not appear when P(O) is low has been reported in the past, both in observational tasks [34] and in active tasks like the one used in this article [20], and suggests that the outcome- and the cue-density biases are not symmetrical. Rather, a high P(O) setting is needed in order to reliably observe the cause-density bias. However, this conclusion again must be cautious because here we were manipulating the base-rate via a pretraining phase, and therefore we lack the optimal conditions to make a fair comparison (see the references for properly controlled tests of this hypothesis).

Additionally, the trial-by-trial data were used to compute the predictions made by the ΔP and the Power PC indexes (Table 5). As in Experiment 1, these indexes did not correlate with the judgments (all *p*s > 0.123), suggesting again that causal judgments were not directly based on the contingency information as computed by these two theories.

**Table 5:** Indexes obtained for the two models, ΔP and Power PC, computed from the actual training data in Experiment 2. The models cannot be computed for some participants (due to division by zero errors), so these cases are removed.

		Control group				Low Base-rate group		
Model	*n*	*M*	*SD*	95% CI	*n*	*M*	*SD*	95% CI
ΔP	64	-0.02	0.21	[-0.07, 0.04]	62	0.01	0.17	[-0.03, 0.06]
Power PC	62	-0.03	0.24	[-0.09, 0.03]	61	0.004	0.21	[-0.05, 0.06]

#### Evidential value questions

**Fig 6:**
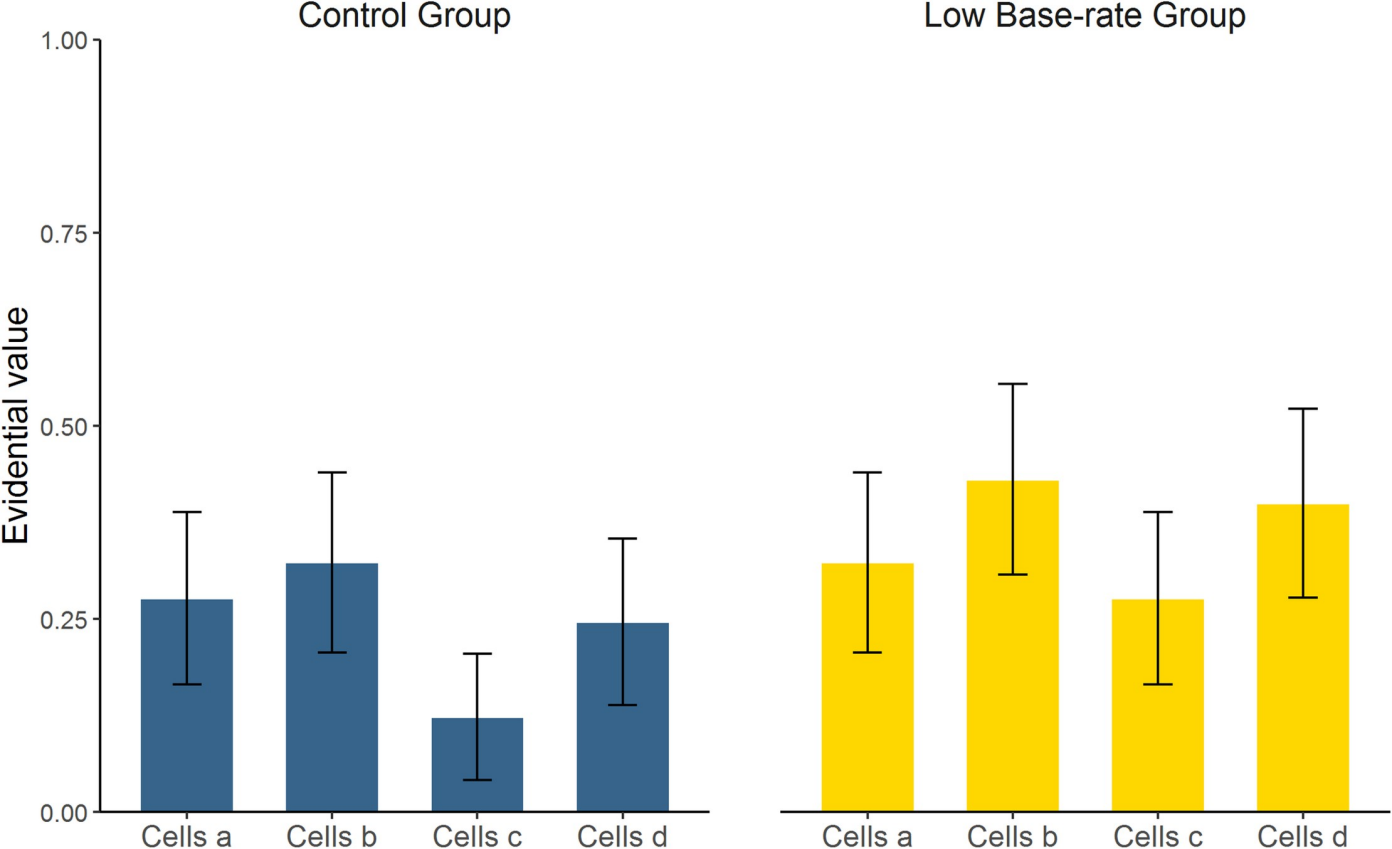
Mean proportion of “evidence” answers to the evidential value questions in Experiment 2, by type of trial: cells *a*, *b*, *c*, and *d*. Error bars depict 95% CIs for the mean.

Finally, Fig 6 depicts the proportion of cells classified as "evidence" in the evidential value questions. These data were analyzed by means of a GLM identical to that in Experiment 1. The model converged at AIC = 608.7. Only the effect of trial type was significant (*p* = 0.014), although the main effect of Group was close to significance level (*p* = 0.055), indicating that, overall, all cells were more likely treated as coincidence in the Control group than they were in the Low base-rate group. However, there was no interaction (*p* = 0.557). See Table 6 for details. Post-hoc contrasts revealed that no trial type differed between the two groups (all *p*s > 0.148). Type *c* trials tended to be the least likely to be categorized as “evidence”, but this was significant only in comparison to *b* trials (*p* = 0.045), so this does not seem a solid result.

**Table 6:** Evidential value questions in Experiment 2: Marginal estimated means (logit scale).

Group	Trial Type	Marginal estimated mean	95% CI
Control	a	-1.068	[-1.712, -0.424]
	b	-0.830	[-1.442, -0.197]
	c	-2.173	[-2.999, -1.347]
	d	-1.246	[-1.910, -0.583]
Low base-rate	a	-0.830	[-1.452, -0.207]
	b	-0.306	[-0.902, 0.290]
	c	-1.078	[-1.721, -0.434]
	d	-0.450	[-1.051, 0.150]

## General discussion

Previous research suggested that prior knowledge and expectations can greatly impact the output of learning processes [14,16,36–38]. The present two experiments show that the expectation of a particular piece of information, the outcome base-rate, is able to modulate the illusion of causality in a standard contingency learning task. When participants believed that the outcome would appear with high base-rate (Experiment 1), then the illusion of causality that is normally observed (and was indeed found in the Control group) was reduced. Conversely, when participants believed that the outcome would appear with a low base-rate (Experiment 2), the illusion of causality became strong even in a situation (i.e., low probability of the outcome) in which it is typically weak or unobserved [13,34].

In these two experiments, the pretraining phase can be seen as a way to induce expectations and beliefs prior to the actual, standard contingency learning phase. The effect of prior knowledge and beliefs on contingency learning has been studied in the past. Notably, people's prior knowledge has been shown to modulate contingency and causal learning. For instance, although contiguity between cause and effect is one of the cues to infer causality, prior knowledge about the potential causal mechanism can modulate it so that a noncontiguous stimulus is perceived as causal [36]. A more systematic investigation about the interplay between prior knowledge and contingency learning was reported by Fugelsang and Thompson [16]. In three causal learning experiments, these authors manipulated two factors, (a) the plausibility of the potential causes prior to the experiment (e.g., eating a food item is a plausible causal candidate of allergic reactions, while doing homework is not), and (b) the actual contingency between the potential causes and the outcomes. They found, in line with White's [39] previous observations, that the participants' judgments were more likely based on the contingency when the causal candidate was plausible than when it was implausible. That is, pre-existing beliefs were able to modulate the extent to which contingency was used in the participants' judgments. The novelty of our experiments with respect to previous ones is that the prior beliefs concerned a very specific attribute of the causal learning situation, namely, the expected outcome base-rate. This attribute was also easily manipulated during the experimental session, by means of a pretraining phase. We used a completely unusual cover story (aliens, mutations) to alleviate any pre-existing bias caused by prior knowledge external to the experiment. Although some previous works investigated manipulations of base-rate, most of them were designed to produce different predictions from two popular competing models, namely ΔP and Power PC [18], so they do not allow to investigate the effect of base-rate manipulations on the causal illusions, which implies using null contingencies. Note that in our experiments both models produce similar predictions (see the Results sections above).

It is worth commenting the overall deviance of the current pattern of results with respect to the most common standard indexes of contingency. Our analyses showed that people’s judgments of causality do not conform to the predictions of the ΔP model or the Power PC model. The same conclusions were found when we computed the predictions by these models from the conditional probability questions (i.e., “perceived contingency”) and from the actual trial-by-trial data (i.e., “actual contingency”). Our participants seem to use information in a way that is not directly captured by these two models.

Next, we describe several ways in which the results of our two experiments might be accounted for. As we will argue, all the following explanations offer partial or imperfect fit to our findings, but the last one in being discussed is flexible enough to account for the whole set of results.

First, we will discuss the associative learning account, which has been traditionally proposed as a suitable general framework for causal learning. Most associative models assume that, during the whole learning session, the context plays a role as a potential alternative cause that stays in the background. This means, among other implications, that (a) whenever the potential cause is not present, the outcome occurrence can be attributed solely to the context, and (b) the context can compete with the potential cause in predicting the outcome. From this perspective, our pretraining phases, in which the target potential cause (i.e., mutagenic agent) was not presented, can be understood as contextual training phases. In Experiment 1, this means that, by the end of the pretraining phase, the context would have acquired some excitatory associative strength, as it has been repeatedly paired with the outcome (70% of the trials). Then, during the subsequent training phase, the context would have been a relatively strong competitor of the target cue, leading to lower causal judgments in the pretrained group than in the Control group. That is, the context, given its previous association with the outcome, could block further learning about the target potential cause [40]. Thus, blocking is a commonly observed learning phenomenon, predicted by the associative theory, that can easily explain what happened in Experiment 1. However, it is more difficult to apply the same explanation to Experiment 2, in which the pretraining phase took place with a low base-rate schedule, and the result was in the opposite direction to Experiment 1 (i.e., previous contextual training led to higher, not lower, causal judgments for the target cause). In principle, and without additional assumptions, the associative theory would predict a weaker blocking effect (or no blocking effect whatsoever) in Experiment 2, but not the opposite result (stronger judgments in the pretrained group).

Additionally, certain associative theories propose that the learning rate parameters of the potential causes (i.e., associability, or salience) can change as a result of previous learning or exposure to the stimuli [41,42]. Thus, the pretraining phase in Experiment 1 (high outcome base-rate), in which the context is repeatedly paired with the outcome, could gradually increase the attention to the context, as in Mackintosh’s theory [41], which in turn could reduce further learning about the potential cause during the training phase. However, again, this argument cannot be directly applied to Experiment 2 to explain the results in the opposite direction (at least without making additional assumptions): why should pretraining the context with low outcome base-rate lead to stronger, not weaker, association with the potential cause, as compared with a Control group with no pretraining at all? In sum, the two associative accounts discussed here cannot completely explain the results of both Experiments 1 and 2, at least without making additional assumptions.

The rest of the potential explanations of our results are not based on associative theories, but on the computational constraints of the rules that could be applied to judge causality by combining different pieces of information (e.g., the ΔP and similar rules). One potential explanation is that the sensitivity of the causal judgments to the base-rate manipulation could be due to a difference in the perception of the conditional probabilities of the outcome (given the cause and the absence of the cause), the two pieces of information that are contrasted in the ΔP rule (Eq 1). Note that the pretraining phase represents, in fact, additional exposure to the probability of the outcome given the absence of the cause, P(O|¬C), i.e., the outcome-base rate. Thus, the difference in the judgments could be due to participants in the experimental groups in both experiments having extra experience with one of the two conditional probabilities involved in the computation of contingency, and therefore higher chances of capturing this information accurately. However, this explanation seems to fall short to reproduce our results, given that we collected conditional probability judgments, and they were accurate in capturing the actual contingencies: in all conditions of both experiments, these judgments were close to the actual programmed values of these probabilities, irrespective of the amount of experience with each piece of information (see Tables 1 and 3). It seems that conditional probabilities were accurately estimated, whereas causal judgments were biased. This is in line with some theories proposing that biases emerge at the moment of combining the acquired information to make the judgment, rather than during the encoding/learning process [30,43]. Nonetheless, one must bear in mind that, in our experiments, these conditional probability judgments were requested after the causal and confidence judgments, which could have contaminated the result.

A closely related possibility to partly explain the results is based on the cause-density bias that we mentioned above: the higher the P(C) (i.e., the more cause-present trials a participant is exposed to), the stronger the overestimation of null contingency [30,35]. In fact, if pretrained participants somehow merged the two phases (pretraining and training), despite being separated by several screens including a judgment and further instructions, they would indeed be experiencing a larger amount of cue-absent trials (cells *c* and *d* in Fig 1) than those participants in the control groups, and this might bias contingency estimations. Crucially, the cause-density bias could explain the results of Experiment 1 (higher judgments in the group with more exposure to cause-present trials), but not those of Experiment 2 which show the opposite pattern.

Additionally, recall that we computed the amount of cause-present trials during the training phase, or P(C). Since participants were free to use the mutagenic agent (i.e., introduce the target cause) as often as they wished, if there were systematic differences between groups in the amount of cause-present trials during the training phase, these could still explain the results. We showed that this was not the case. In both experiments, the groups without pretraining showed the expected behavior concerning this variable: in Experiment 1 (high probability of the outcome), there was a positive correlation between P(C) and judgments (i.e., a cause-density effect), while in Experiment 2 (low probability of the outcome), this correlation was absent, as we found in previous studies [20] [34]. Moreover, in the pretrained groups, we found no correlation between P(C) and judgments in either experiment, which suggests that the final causal judgment seems to be affected directly by our expectation manipulation rather than by other factors that are introduced later during the training phase, like the number of cause-present trials. This is also in line with the abovementioned accuracy of the answers given to the conditional probability questions in all groups: biases in the causal judgments appear to be dependent on assumptions made before the training phase starts.

A further possibility to explain the results concerns the plausibility of the causal candidate. As we discussed above, several studies have tried to identify the conditions that determine when the contingency is relevant for causal judgment [16,39], concluding that contingency information can be sometimes overridden by other cues. One of the factors that matter is the a priori plausibility of the causal relationship (e.g., eating a food item is a more plausible cause of allergy than is doing homework): previous research suggests that, in the extreme case, if the plausibility is low, participants can ignore the contingency data. In our Experiment 1, the high base-rate pretraining could have promoted the belief that additional, hidden, causes were operating. Then, if hidden causes seem a plausible causal candidate, participants might discount to some extent the role of the target cause, by virtue of the so-called “logic of exoneration” that is characteristic of most rational accounts of causal reasoning [44]. That is, if one potential cause or hypothesis increases its plausibility, the rest of potential hypotheses are reduced accordingly. Therefore, the judgment would be reflecting a priori plausibility of the target cause rather than the actual experience acquired during the training phase. In line with this idea, some computational models of causal inference [45] propose that people bias their causal learning processes by making a priori assumptions that are related to the plausibility of potential alternative causes. For example, it has been proposed that people display a preference for simple models (i.e., as few causal candidates as possible) with either very strong or null causal effects. This assumption has been described as a “strong and sparse prior” [45]. According to this assumption, one can predict that, when a hidden cause is repeatedly predicting the outcome (i.e., pretraining phase in Experiment 1), people assume that it is unlikely that other causes could also produce the outcome (i.e., they do not tend to consider additional causes if unnecessary), which explains why they are reluctant to give high judgments to the target cause. In Experiment 2, we can apply the same logic: in the pre-training phase, the hidden causes seem to produce little effect (i.e., there are few outcome occurrences), and therefore people are willing to accept that other causes can play a role, in contrast with Experiment 1. However, this rationale is insufficient to explain why participants in the Low base-rate group gave higher, not equal, judgments to the target cause, compared to the Control group without pretraining. Therefore, the explanations based on a priori assumptions about plausibility or simplicity cannot completely account for the results of the two experiments.

Finally, we describe a last explanation that seems suitable for the whole set of results that we have reported here. This explanation is based on the popular assumption that the different pieces of information that are combined to produce a causal judgment are given different degrees of evidential value, or subjective weights. Trial-weighting mechanisms have been proposed to underlie many deviations of contingency judgments from the normative contingency values as given by the ΔP index [46–48], which has given birth to weighted versions of the ΔP rule that include a weight parameter for each trial type. Additionally, many other theoretical models of contingency learning can be adapted to feature trial-weighting mechanisms. For instance, the Rescorla-Wagner model [49] can instantiate such mechanism by including different values for their two free parameters, alpha and beta [34].

Typically, the rank of subjective trial weights that has been documented in the literature is *a* > *b* ≥ *c* > *d* [29,50–52], which would only explain the results of those conditions in which we found an illusion of causality (i.e., Control group in Experiment 1, low base-rate in Experiment 2). To account for all the results presented here, the trial weights ranks should be different for each condition. In particular, what we need to assume is that the prevalence of type *c* trials in the pretraining phase of Experiment 1 should reduce the influence of outcome-present trials (i.e., *a* and *c*) relative to outcome-absent trials (*b* and *d*) in the subsequent training phase. Applying the same rationale, the inverse result would be found in Experiment 2: the low proportion of type *c* cells in the pretraining phase could increase the weight of subsequent outcome-present trials relative to outcome-absent trials when making the judgment. Are there any extant models endowed with the capacity to make these predictions?

In this regard, associative models such as Rescorla-Wagner [49] offer additional flexibility over non-associative or rule-based models [47] because the weights are determined by the combination of their four free parameters (alpha and beta of the cue, the outcome and the context), and additionally because these parameters can change as a result of previous experience [41,42]. However, some have pointed out that arbitrary changes in the associability parameters are not justified between conditions in which the same materials and cover story have been used [53,54], which somewhat limits this flexibility. Importantly, no previous theory has proposed explicitly that the weights are dependent on prior knowledge about outcome-base rates. Rather, these weights tend to be considered as fixed or stable, despite evidence that some manipulations (e.g., goal-driven reasoning) can overcome the habitual neglect of *d* cells [55].

However, a rational analysis of the informativeness of each trial type offer predictions that actually align with our results. MacKenzie and Mikkelsen [17] provided such analysis, according to which, if certain assumptions are met, some types of trials are more informative than others when judging potential causal relations. If the stimuli involved in the task (i.e., the potential cause and the outcome) are assumed to occur with low probability, then their joint occurrence (trials *a*) should be given the most weight, while their joint absence (trials *d*) should be treated as the least important, with trials *b* and *c* falling in between. That is, assuming rarity of the cause and outcome, the mentioned trial weight rank *a* > *b* ≥ *c* > *d* is normatively correct. This would be the case of our Experiment 2, in which we pretrained participants to believe that the outcome was a rare event, and then the illusion of causality appeared. On the other hand, we can apply the same logic in the opposite direction. Assuming that the cause and the outcome would occur very frequently would lead participants to a different trial weight rank in which type *a* trials are less prominent. This would explain the results of our Experiment 1, in which pretraining in a high outcome base-rate background led to lower illusion of causality. Thus, the prediction from a rational account of information usage [17] fits well with our two experiments simultaneously, in contrast with the rest of the accounts that we have described, which offer only partial solutions.

So far, we have focused on our main dependent variable, causal judgments. The results concerning our evidential value questions were not completely clear, although they are partially in line with the causal judgments, and thus deserve a comment in this section. These questions were intended as a rather direct measure of the subjective evidential value granted to each trial type (i.e., the trial weights that we mentioned in the previous paragraph). In Experiment 1, the Control group was exposed to the typical conditions in which the causal illusion appears (i.e., high probability of the outcome) [13,34]. Thus, we would expect that in this group the most important trials would be those that support the causal relation, that is, *a* and *d*, and this is what we found, although the results were not significant (only *c* trials were consistently categorized as “coincidence”). To be consistent with causal judgments, in the High base-rate group we would expect that *a* and *d* trials should be less important than in the Control group. Our data show that all but *b* trials were significantly categorized as “coincidence” in the High base-rate group. Then again, this could not be confirmed because the results of the omnibus analyses were nonsignificant. In Experiment 2, the effects of trial type and group were significant (the latter only marginally, though). The ordered rank of trial types was consistent between groups, indicating a prevalence of type *b* and *d* trials (or, more clearly, a tendency to treat *c* trials as “coincidence” more often than the rest of trials). However, we found no significant differences between trial types (except for *b* vs. *c*), so we refrain from drawing conclusions. When interpreting the evidential value answers, one must keep in mind that the amount of trials of each type was different for each experiment and also for each participant, given that they were free to choose when to use the cause. This different exposure to the trial types could have influenced the subsequent measure. In addition, since the evidential value questions were not planned as our main dependent variable, they were collected with exploratory aims at the end of the session. Therefore, they could have also been contaminated by the previous tasks. Finally, as they were collected as a binary response, they seem not to be sensitive enough to be informative in this design. In sum, this variable yielded no conclusive results. Further studies could consider assessing the relative importance of each trial type with more sensitive measures (e.g., ratings) instead of dichotomous responses, while trying to control for extraneous variables.

Another limitation of our studies can in fact be applied to the vast majority of contingency-learning experiments. In this standard procedure, causes and effects are binary (either they occur or they do not), and time is discretized into trials. Thus, participants can only observe whether there is a joint occurrence of the potential cause and the outcome within a given trial (i.e., a particular moment in time). There is no possibility, hence, to observe the temporal dynamics of causality. For example, it could be that the mutagenic agent actually works to produce the mutation, but it needs some time before the effect is observed, and therefore we observe a seemingly null contingency. Making this type of assumptions can also distort the way base-rates are interpreted [56]. For example, observing a long period of time in which nothing happens (i.e., cells *d*) might induce the expectation of a very low base-rate of all events. Currently, we have no way to gain insight into this question without using a completely different procedure that actually involves time.

## Conclusions

To sum up, we have reported two experiments in which the outcome base-rate expectations were successfully manipulated in opposite directions (Experiment 1 vs. Experiment 2). These expectations led to a modulation of the causal illusion in the causal judgments that is compatible with a rational account of cell information usage [17] that translates to differential weighting of the four trial types when estimating causality. Further research should disentangle the role of cell-weighting mechanisms in the causal illusion.

The contribution of our experiments is not only to advance in our understanding of the basic cognitive processes involved in causal judgment. Rather, they can provide insight to develop certain applications. Previous research has proposed that causal illusions such as those reported here entail both good and bad consequences for people [57]. On the one hand, the causal illusion could underlie many every-day irrational beliefs, attitudes and behaviors: pseudomedicine usage, paranormal beliefs, pathological gambling, and superstitions [2,7,58–60]. These practices can even be dangerous (e.g., when one person resorts to a pseudomedicine, abandoning a valid treatment). On the other hand, certain types of biases, including the causal illusion, seem to be associated to optimism and positive attitude: for example, causal illusions are apparently weaker in depressed people [61,62], although see [63,64]. Therefore, it would be desirable to design ways to bring the causal illusion under our control, to prevent it when it leads to harmful behaviors [65], and to promote it when it fosters wellbeing [66]. In this regard, our two experiments illustrate a means to either prevent causal illusions (Experiment 1) or to enhance them (Experiment 2) by shaping the assumptions that people make before collecting the contingency information. That is, one potential application of Experiment 1 to prevent pseudomedicine usage would be to show that certain health conditions (precisely those that are frequently treated with pseudomedicine, such as homeopathy) possess, in fact, a large chance of spontaneous remission (e.g., back pain, headache). In other words, they have a high outcome base-rate. Thus, when one patient knows that the remission base-rate is high without taking any treatment, and decides later to try a pseudomedicine, he/she would be less likely to develop a misleading causal illusion, just as our participants in Experiment 1. Conversely, the rationale of Experiment 2 can be used to take advantage of base-rate knowledge in those situations in which a causal illusion is positive. For example, it could be applied to the academic context to strengthen self-confidence in students who aim to get difficult-to-obtain or unlikely outcomes (e.g., prizes and other indicators of academic success): if they are first aware of the low base-rate of these events, they will more likely attribute their occurrence to their efforts and actions when they eventually experience such events (as in Experiment 2).

## Supporting information

S1 Appendix Supplementary analyses. Bayesian update of the base-rate beliefs. We use simulations of the beta distribution to model the update of base-rate knowledge, given the information from the pretraining and training phases.
(PDF)
