# Supplementary material for: Base-rate expectations modulate the causal illusion
Source: PLoS One. 2019 Mar 5;14(3):e0212615. doi: 10.1371/journal.pone.0212615 (PMC6400408; doi:10.1371/journal.pone.0212615)
Supplement: S1 Appendix — Bayesian update of the base-rate beliefs. We use simulations of the beta distribution to model the update of base-rate knowledge, given the information from the pretraining and training phases. (PDF) [file pone.0212615.s001.pdf]

S1. Supplementary Analyses

Bayesian update of the base-rate knowledge: Experiment 1.

In our design, we asked about the base-rate knowledge in two occasions: (1) prior to the training phase, and (2) after the causality and confidence judgments, i.e., the base-rate question and the P(O|¬C) question, respectively, using exactly the same wording. This allows us to gain insight into the question of how people use the information acquired during the pretraining and training phases to update their base-rate knowledge, in a Bayesian fashion. The logic of this analysis is as follows. In the Bayesian updating framework, a prior distribution, P(H), is combined with the likelihood of the observed data, or P(D|H), to obtain a posterior distribution, P(H|D). Here, the parameter over which the inference is carried out is the base-rate of the outcome, P(O|¬C). Thus, we assume that people try to update their belief about the outcome base-rate based on the data they see during the pretraining phase P(H|D). Because the occurrence of the outcome is a discrete, binary event (either it occurs, 1, or not, 0), the whole process can be modelled by means of beta distributions, which are tractable and allow us to make the computations. Although the following analysis remains speculative and rests on assumptions that could be wrong, we include it for completeness.

I. Updating beliefs with data of the pretraining phase. There are two approaches we can take to gain insight into the belief update process. First, we ask whether people in the pretrained group (high base-rate group) are rationally updating their base-rate knowledge with the information that we provided them during the pretraining phase (i.e., it could be an indirect way of assessing the success of the pretraining manipulation). Since participants in the high base-rate group were not asked about their base-rate expectations before the pretraining phase, we can use the base-rate judgments given by participants in the Control group to have an idea about the overall "prior distribution" of base-rate knowledge, before any information is given. Thus, we took the distribution of the base-rate judgments from the Control group, and estimated the parameters of a beta distribution (Figure S1, left panel) by using the MASS package for R [1,2]. Before fitting the function, the judgments were rescaled to range from 0 to 1. The figure is, thus, a beta approximation to the actual data provided by participants from the Control group in the base-rate question. Note that the beta approximation takes as input the distribution parameters (mean,

variance) of the aggregation of all the judgments in the Control group. Ideally, we would have obtained instead a distribution for each individual, but this is impossible because they were asked about the a priori base-rate expectation only once (i.e., we had only one data point per participant). Thus, our analysis rests on the (probably wrong) assumption that there are no essential individual differences in the shape of the prior belief distributions, and that therefore the mean of the whole group mirrors well the beliefs of individuals.

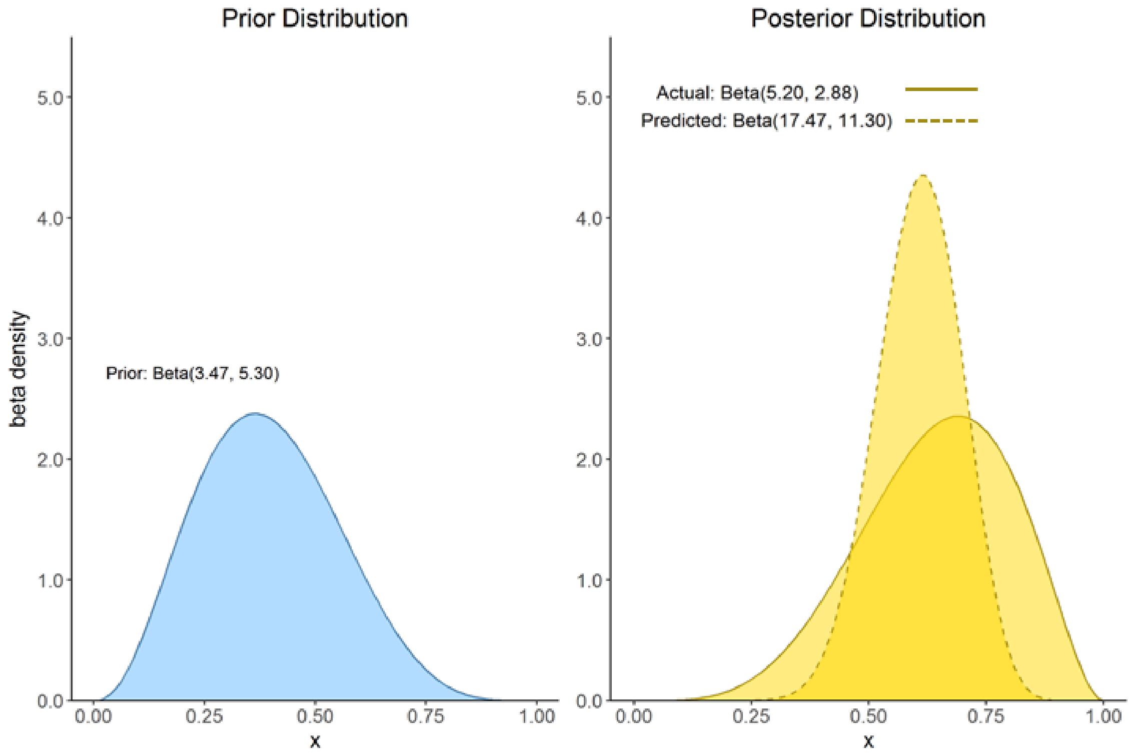

Figure S1. Bayesian update of the base-rate information in Experiment 1, before and after the pretraining. The parameters for the beta distributions are depicted on the figures.

The distribution in Figure S1 (left panel) is shifted to the left because many participants assume that the base-rate of a mutation is below 0.50, which is consistent with the observation (mentioned in the main text) that lay people commonly assume a low base-rate for mutations. Next, we will assume that all participants (including those in the High base-group) would possess a belief distribution similar to the one here depicted before any information or pretraining is given, although in the High base-rate group we did not ask before the pretraining.

Now, this prior distribution, P(H), can be updated with the information provided in the pretraining phase (i.e., the data D), which consists of a sequence of 14 mutant aliens

("outcomes") and 6 non-mutants ("no-outcomes"). Both quantities can be interpreted in terms of the shape parameters of the beta distribution as α and β, respectively. Thus, the predicted posterior distribution, P(H|D), according to a normative Bayesian update is depicted in dashed line on the right panel of Figure S1, and possesses parameters $\alpha_{prior}+14$ and $\beta_{prior} + 6$. As one can imagine, this predicted posterior distribution pushes the probability distribution to the right, because it somewhat contradicts the prior beliefs (i.e., the mutants are not rare, but actually quite frequent, 16 out of 20 observations). Consequently, the predicted posterior (dashed line) is centered on values above 0.50. This predicted posterior is the normatively expected distributions of beliefs in the High base-rate group after pretraining, assuming that their initial prior beliefs were similar to those obtained in the Control group.

Finally, we depict on the same panel of Figure S1 (in solid line) the fitted beta distribution obtained from the base-rate judgments in the High base-rate group. This line was computed by fitting a beta distribution to the collection of actual base-rate judgments collected after the pretraining in the High base-rate group. This implies again the (not granted) assumption that group-level aggregated distributions correspond well to individual beliefs. Overall, the shift of the distribution from the left panel to the right panel of Figure 2 indicates that the pretraining manipulation worked to increase the assumed base-rate of the outcome in the High base-rate group. The overlap between the predicted and the actual (empirical) distributions in the right-hand panel tells us that people in the High base-rate group correctly updated their prior beliefs about the base-rate, according to a normative Bayesian update, or at least they moved their beliefs in the right direction. If anything, they seem to show more uncertainty in their estimation than should be expected. However, we should interpret the spread of these distributions with caution, as we are observing densities that are fitted from aggregated data that likely do not capture well the uncertainty of individual participants. This is a limitation imposed by the fact that we have only one data point for each individual, as the experiment was not designed with this analysis in mind.

II. Updating beliefs with data of the training phase. The second question we ask is whether participants were able to integrate the information from the training phase to eventually produce accurate P(O|¬C) judgments at the end of the session. We proceed in the following way. First, Figure S2 (left panel) shows the fitted beta distributions obtained

from the base-rate judgments, which were asked before the training phase and work now as our prior distributions. Note that in this second analyses we are presenting a beta approximation to the distribution of the judgments in the two groups: In the High base-rate group, the base-rate judgments were collected after the pretraining with a high base-rate, whereas in the Control group, these judgments were collected without any prior information. These will be our prior distributions, P(H), which will be updated with the information acquired during the training phase to obtain a posterior distribution, P(H|D).

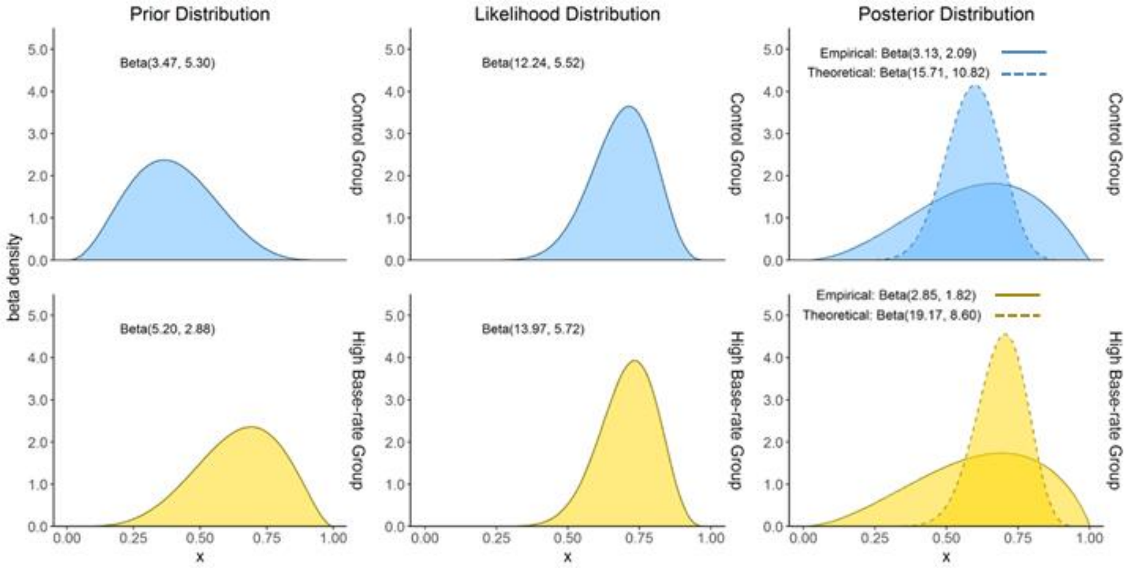

Figure S2. Bayesian update of the base-rate information in Experiment 1, before and after the training phase. The parameters for the beta distributions are depicted on the figures.

The fitting to beta distributions was carried out in the same way as in the previous approach, hence it has the same limitations. By looking at the prior distributions (Figure S2, left panel), which are obtained from the base-rate judgments collected before the training phase, we can observe again how the pretraining phase shifted the distribution towards higher values in the High base-rate group, compared to the Control group.

In the middle column, we depict the likelihood distribution, P(D|H), for each group. These are computed from the actual sequences of trials produced by each participant, and then averaged by group. Specifically, we counted the number of outcome occurrences (trials *c*) and the number of outcome non-occurrences (trials *d*) for each participant, then averaged these numbers by group, and finally assigned them to the α and β parameters of

the beta distribution for the likelihood function depicted in the middle column. They were very similar in the two groups, as the training was almost identical.

The right-hand column includes two pieces of information. First, in dashed line, we present the beta distribution resulting from combining the prior distribution with the information of the training phase according to Bayes' rule, which we call the "theoretical" posterior distribution. Since we are working with beta distributions, this computation is easy, as the parameters of the theoretical posterior are: α_{prior} + number of outcome occurrences (trials *c*), and β_{prior} + number of outcome non-occurrences (trials *d*). As we can observe, the rational Bayesian update predicts little differences between groups, since they are seeing essentially the same information during the training phase and only differ in their priors.

Finally, in solid line, we can see the "empirical" posterior distribution, which is obtained from the actual judgments answered to the P(O|¬C) question. They do not overlap much with their respective theoretical distributions, because participants seem to produce a wide range of values in response to this question, although the empirical distributions are centered on the expected values (above 0.50) in the two groups. However, we must be cautious when interpreting the spread of the distributions, since we obtained them from aggregated, group-level data, instead of from individuals (which was impossible in this design with only one data point per participant). Thus, the spread in these distributions could reflect not the uncertainty in the parameter estimation due to different participants having radically different beliefs, but rather the random variability that is produced by each individual producing different judgments despite sharing a common belief on the parameter, which they report in their answers with some random noise or variability. This is a limitation of the way we conducted these simulations. Still, without interpreting the shape of the distribution, the centrality parameters suggest that people captured well the base-rate information during the pretraining and training phases (and this aligns with the main analyses).

Bayesian update of the base-rate knowledge: Experiment 2.

As we did in Experiment 1, we conducted an analysis of the Bayesian update process of the base-rate knowledge in two different moments. First, from before the pretraining phase

(i.e., base-rate judgments in the Control group) to after the pretraining phase (i.e., base-rate judgments in the Low base-rate group).

I. Updating beliefs with data of the pretraining phase. Figure S3 shows this update process. Base-rate judgments in the Control group are taken as a prior distribution, and they show (as in Experiment 1) that people spontaneously assume that mutants should appear with relatively low base-rate (i.e., the prior peaks below 0.50). Then this prior is updated with the pretraining information, which in this case indicates a low base-rate of the outcome (i.e., 6 mutants and 14 non-mutants), which gives us a predicted posterior (dashed line) with parameters $\alpha_{prior} + 6$ and $\beta_{prior} + 14$. Finally, we also depict the fitted beta distribution from the aggregated, actual base-rate judgments in the Low base-rate group (solid line). There is a generally large overlap between the two distributions. Note particularly the central tendency of both distributions: they shift to the left because the pretraining induces the belief that mutations have low base-rate.

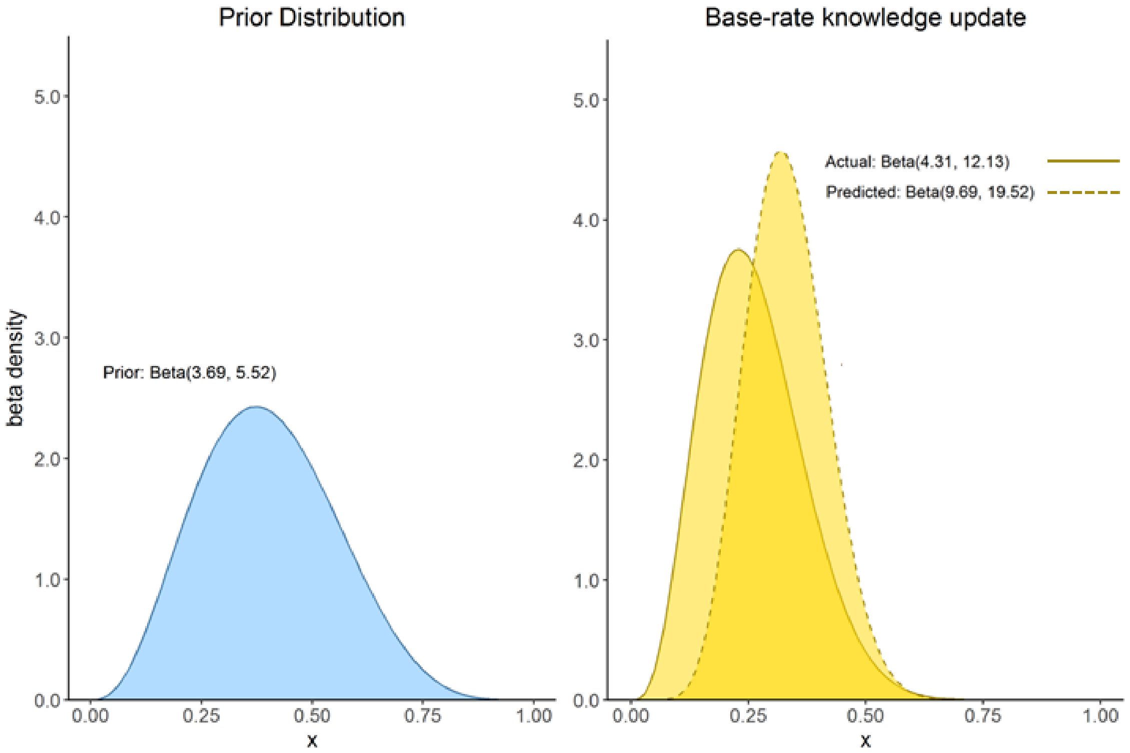

Figure S3. Bayesian update of the base-rate information in Experiment 2, before and after the pretraining. The parameters for the beta distributions are depicted on the figures.

II. Updating beliefs with data of the training phase. Secondly, we carried out an analyses of the base-rate knowledge update process from before the training phase to after the training phase, as in Experiment 1. Figure S4 depicts the resulting distributions. First, we observe the expected differences in the prior distributions (computed from the base-rate judgments right before the training phase): although both groups assume that mutants appear with relatively low base-rate, this tendency is clearer for pretrained participants, who are better informed about this base-rate. Then, the middle column represents the likelihood distributions when observing the actual sequences of trials in the training phase. Specifically, we counted the number of "c" and "d" trials, which are relevant for the base-rate computation, averaged them by group, and assigned them to the two parameters of the beta distribution. Finally, the right-hand column depicts both the "theoretical" posterior resulting from the combination of the prior and the training information, and the "empirical" posterior, which is obtained from the actual judgments provided to the P(O|¬C) question. As in Experiment 1, the empirical posteriors correctly move in the right direction (i.e., towards lower values of outcome base-rate). The spreads of the empirical and theoretical distributions differ noticeably, but we have mentioned before that these spreads cannot be interpreted as participants showing a larger range or a more variable distribution of beliefs, because we did not estimate the beta parameters from individual responses, but from aggregated, group-level data, which imposes limitations.

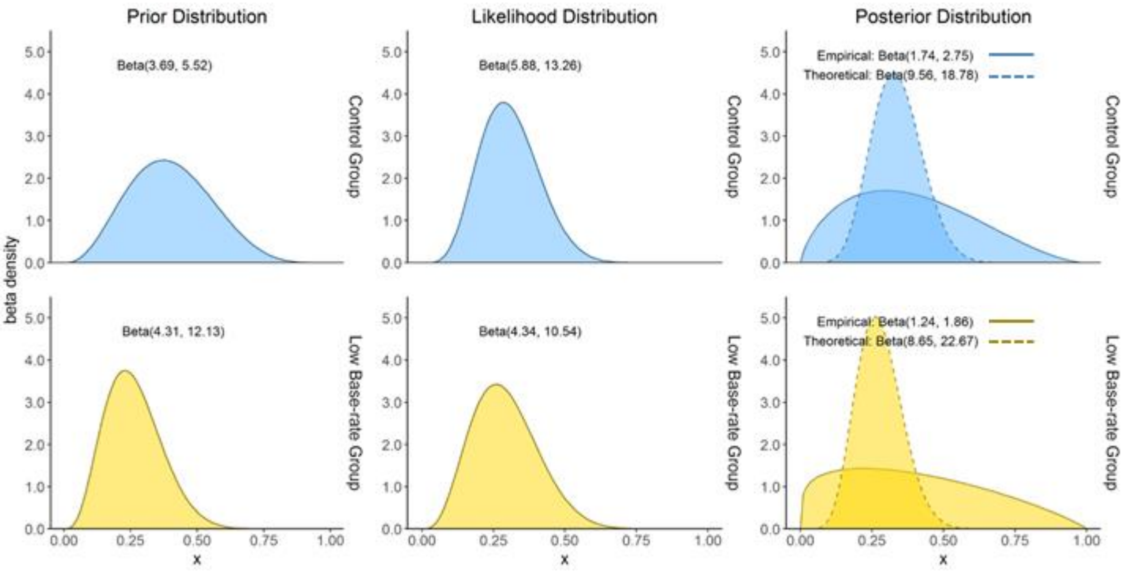

Figure S4. Bayesian update of the base-rate information in Experiment 1, before and after the training phase. The parameters for the beta distributions are depicted on the figures.

As in Experiment 1, we must insist in that the analyses reported in this Supplementary Analyses file are based on assumptions that could be wrong. Particularly, for assuming that parameters fitted from group-level data distributions capture accurately the beliefs of individuals, two conditions should be true: (a) that individuals are quite homogeneous in their belief distributions, that is, that all participants in the group share a common value for the parameter, and (b) that the spread of the distribution observed at the aggregated group level is due to each individual reporting a judgment that reproduces this common parameter, with some random variation. If these two conditions do not hold (and they probably do not), then the inference on these analyses will be seriously limited, especially when interpreting the shape or spread of the distributions. However, we report the belief update analyses because, when the central tendencies of the distributions are taken together with the rest of results reported in the main text, they show a coherent, convergent conclusion (i.e., pretraining works to induce high/low base-rate expectations, people match the probabilities they are fed through the training phase), and thus we think that, although imperfect, our approximation is good enough to shed some light on the belief update process.

References

1. Ripley B, Venables B, Bates DM, Hornik K, Gebhardt A, Firth D. Package 'MASS.' 2018.

2. Venables WN, Ripley BD. Modern Applied Statistics with S. 2002.
